# pH-Induced Transition Between Single-Chain Macrocyclic Amphiphile and [*c*2]Daisy Chain-Based Bola-Type Amphiphile and the Related Self-Assembly Behavior in Water

**DOI:** 10.3389/fchem.2019.00894

**Published:** 2020-01-24

**Authors:** Pi Wang, Ruihuan Wang, Danyu Xia

**Affiliations:** ^1^Ministry of Education Key Laboratory of Interface Science and Engineering in Advanced Materials, Taiyuan University of Technology, Taiyuan, China; ^2^Scientific Instrument Center, Shanxi University, Taiyuan, China

**Keywords:** macrocyclic amphiphile, stimuli responsiveness, daisy chain, self-assembly, pillararene

## Abstract

Macrocyclic amphiphiles, a type of amphiphiles synthesized based on macrocyclic compounds, have attracted much attention over the past decades due to their unique superiority in the construction of various functional nanomaterials. The regulation of the state of macrocyclic amphiphiles by introducing stimuli-responsive motif to macrocyclic amphiphiles is an efficient way to extend their applications in diverse fields. Herein, pillararene-based macrocyclic amphiphile **H1** was prepared. **H1** can act as single-chain amphiphile to self-assemble into micelles in water when the pH was ≥5.0. **H1** can be protonated to turn into **H2** when pH changed to <5.0. Interestingly, **H2** formed [*c*2]daisy chain-based bola-type supramolecular amphiphile. This bola-type supramolecular amphiphile self-assembled into nanosheets in water. Therefore, pH-induced transition between single-chain macrocyclic amphiphile and bola-type amphiphile and the corresponding self-assembly system based on pillararene in water were constructed.

## Introduction

Amphiphiles, carrying both hydrophilic and hydrophobic parts connected by covalent bonds, are a class of interesting molecules to fabricate self-assembly systems (Discher and Eisenberg, 2002; Sorrenti et al., 2013; Chang et al., 2019b). Owing to their amphiphilic nature, amphiphiles can self-assemble into various nanostructures in water that can be applied in various areas, including drug/gene delivery, photodynamic therapy, and bioimaging (Zhang and Wang, 2011; Hu et al., 2013; Kelley et al., 2013; Ma and Zhao, 2015; Yu et al., 2015; Ji et al., 2016; Xia et al., 2016; Webber and Langer, 2017; Guo et al., 2018; Zuo et al., 2018; Wang S.-P. et al., 2019; Wang Y. et al., 2019). Amphiphiles synthesized based on macrocyclic compounds, namely, macrocyclic amphiphiles (Jie et al., 2015; Zhu et al., 2018), have gained growing attention in recent years. Compared with traditional amphiphiles, macrocyclic amphiphiles possess unique superiority in the construction of various functional nanomaterials, e.g., the incorporation of functional groups and intriguing properties can be achieved by host–guest interactions without extra additives and tedious synthesis (Wei et al., 2014; Wang et al., 2015; Shulov et al., 2016; Geng et al., 2017; Redondo-Gomez et al., 2019). In addition, the regulation of the state of macrocyclic amphiphiles by introducing stimuli-responsive motif to macrocyclic amphiphiles is an efficient way to extend their applications. Therefore, external stimuli-responsive macrocyclic amphiphiles play important roles in many fields, such as injectable materials, sensing, and cell imaging (Chang et al., 2014; Himmelein et al., 2014; Wang et al., 2015; Yang et al., 2016; Himmelein and Ravoo, 2017; Gao et al., 2018; Hu et al., 2018; Sun et al., 2018; Lee et al., 2019; Li et al., 2019).

**Scheme 1:**
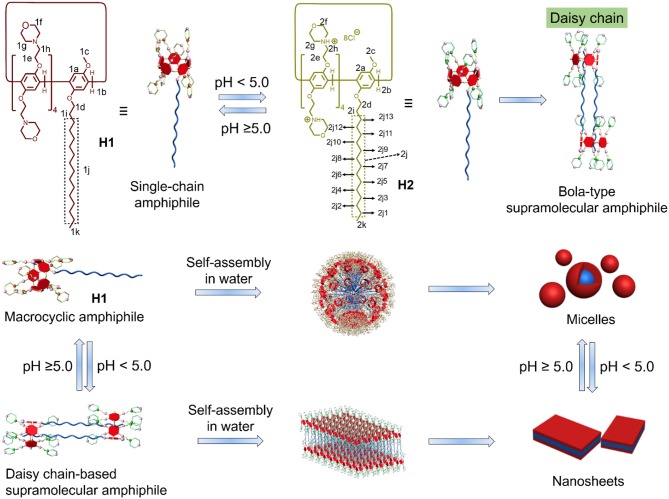
Chemical structures of the macrocyclic amphiphiles **H1** and **H2** and cartoon representation of the [*c*2]daisy chain-based bola-type supramolecular amphiphile and the pH-responsive self-assembly.

Pillararenes, the generation of macrocycles next to crown ethers, cyclodextrins, calixarenes, and cucurbiturils, have been widely studied in the past decade (Li et al., 2017; Ping et al., 2017; Sathiyajith et al., 2017; Hua et al., 2018, 2019; Chen et al., 2019; Xu et al., 2019). Owing to their facile synthesis, easy functionalization and excellent host–guest recognition property, pillararenes have been widely applied to construct amphiphilic self-assembly systems (Shi et al., 2016; Xia et al., 2017; Zhang et al., 2018; Xiao et al., 2019). The rigid and symmetric structure of pillararenes make them good candidates for the construction of macrocyclic amphiphiles. Several types of pillararenes-based macrocyclic amphiphiles have been reported up to now: (1) the non-symmetric pillararenes with half hydrophilic groups and half hydrophobic groups from non-symmetric monomers (Yao et al., 2012; Yu et al., 2013); (2) the difunctionalized pillararene-based macrocyclic amphiphiles from copillar[5]arenes (Gao et al., 2013); (3) monofunctionalized pillararene-based macrocyclic amphiphiles by linking hydrophobic tails to symmetric pillararenes (Jie et al., 2014); and (4) the symmetric *per*-functionalized pillararenes-based amphiphiles (Nierengarten et al., 2013; Chang et al., 2014, 2019a; Yang et al., 2016; Sun et al., 2018). The obtained macrocyclic amphiphiles from these methods displayed interesting stimuli-responsiveness and applications, indicating the importance of pillararenes-based macrocyclic amphiphiles. Herein, we developed a new efficient way to synthesize pillararenes-based macrocyclic amphiphiles. First, we synthesized a long alkyl-containing copillar[5]arene from previous literature. Then, pH-sensitive morpholine groups were covalently linked to the copillar[5]arene to prepare a single-chain macrocyclic amphiphile **H1**. Interestingly, **H1** transformed into protonated state **H2**, which formed [*c*2]daisy chains in water when the pH-value decreased under 5.0. As a result, single-chain amphiphile **H1** turned into bola-type supramolecular amphiphile. Moreover, the pH-responsive self-assembly behavior was investigated. Single-chain amphiphile **H1** self-assembled into micelles in water. When the value of pH decreased to under 5.0, micelles transformed into nanosheets due to the formation of bola-type supramolecular amphiphiles based on the [*c*2]daisy chain structure (Scheme 1).

## Materials and Methods

All reagents were commercially available and used as supplied without further purification. Compounds **a** were prepared according to published procedures (Shi et al., 2014). NMR spectra were recorded with a Bruker Avance DMX 600 spectrophotometer or a Bruker Avance DMX 400 spectrophotometer. Low-resolution electrospray ionization mass spectra were recorded with a Bruker Esquire 3000 Plus spectrometer. High-resolution mass spectrometry experiments were performed with a Waters UPLC H-Class QDA instrument. The melting points were collected on a SGW X-4 automatic melting point apparatus. The determination of the critical aggregation concentration (CAC) values was carried out on a FE38 instrument. Transmission electron microscopy investigations were carried out on a JEM-1200EX instrument. Atomic force microscopy experiments were performed by a Bruker Multi-Mode 8.0 instrument.

### Synthesis of H1

**H1** was synthesized from compound **a** and morpholine (Scheme S1). Compound **a** (1.08 g, 0.622 mmol) and morpholine (0.566 g, 6.50 mmol) were added to acetonitrile (10.0 ml). The solution was refluxed overnight. Then, the crude product was purified by a silica gel column using dichloromethane as eluent (0.421 g, 38%) (Mp: 75.0–77.0°C). The ^1^H NMR spectrum of **H1** is shown in Figure S1. ^1^H NMR (400 MHz, CDCl_3_, 298 K) δ (ppm): 6.85 (s, 10H), 4.14–4.09 (m, 8H), 3.98–3.92 (m, 9H), 3.75–3.73 (m, 47H), 2.88–2.75 (m, 16H), 2.62–2.61 (m, 32H). 1.95–1.86 (m, 2H), 1.84–1.72 (m, 2H), 1.56–1.47 (m, 2H), 1.41–1.32 (m, 2H), 1.16–1.07 (m, 20H), 0.85 (t, *J* = 8.0 Hz, 3H). The ^13^C NMR spectrum of **H1** is shown in Figure S2. ^13^C NMR (100 MHz, CDCl_3_, 298 K) δ (ppm): 149.47, 148.84, 148.77, 127.82, 127.65, 127.50, 127.45, 127.06, 114.32, 114.18, 112.90, 67.69, 65.99, 65.99, 65.81, 57.23, 54.93, 53.27, 52.34, 30.91, 28.78, 28.72, 28.64, 28.59, 28.44, 28.39, 28.35, 28.28, 25.13, 21.68, 13.13. High-resolution electrospray ionization mass spectrometry is shown in Figure S3: *m*/*z* calcd for [M + 2H + e]^+^ C_102_H_158_N_8_O_18_, 1,783.16941, found 1,783.16784, error −0.9 ppm; *m*/*z* calcd for [M + 3H + e]^2+^ C_102_H_159_N_8_O_18_, 892.08862, found 892.08527, error −3.8 ppm.

### Critical Aggregation Concentration Determination

The CAC determination is based on the dependence of the solution conductivity on the solution concentration. Generally, the slope value of the change in conductivity vs. the concentration above CAC is higher than the slope below the CAC. As a result, the CAC-value is the junction of the conductivity–concentration plot. To measure the CAC of **H1** and **H2**, the conductivities of their solutions at different concentrations (from 0 to 0.16 mM and from 0 to 0.25 mM, respectively) were determined. Therefore, through plotting the changes of the conductivity vs. the concentration, the CAC of **H1** and **H2** can be obtained.

### Transmission Electron Microscopy Experiments

The self-assembled structures of **H1** and **H2** were investigated by TEM. A solution of 1.00 × 10^−4^ M **H1** was made first in water. The samples of **H1** were prepared by drop coating this solution onto a carbon-coated copper grid. The solution of **H2** were obtained by adding hydrochloride acid to the solution of **H1**. Then, the TEM samples of **H2** was prepared by drop coating the solution on a carbon-coated copper grid. TEM experiments were performed on a JEM-1200EX instrument.

### Atomic Force Microscopy Experiments

The self-assembled structure **H2** was investigated by atomic force microscopy (AFM). A solution of 1.00 × 10^−4^ M **H1** was prepared in water. The solution of **H2** were obtained by adding hydrochloride acid to the solution of **H1**. Then, the TEM samples of **H2** was prepared by drop coating the solution on a Si substrate. AFM experiments were carried out on a Bruker Multi-Mode 8.0 instrument.

## Results and Discussions

### ^1^H NMR Spectroscopy Experiments

**Figure 1:**
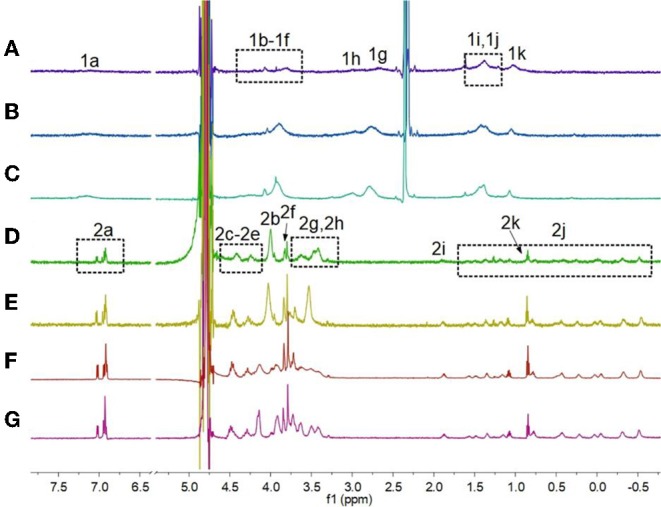
Partial ^1^H NMR spectra (600 MHz, 3:1 D_2_O/CD_3_CN, room temperature) of **H1** (2.50 mM) under different pH conditions: **(A)** pH 7.0, **(B)** pH 6.0, **(C)** pH 5.0; partial ^1^H NMR spectra (600 MHz, D_2_O, room temperature) of **H1** (2.50 mM) under different pH conditions: **(D)** pH 4.0, **(E)** pH 3.0, **(F)** pH 2.0, **(G)** pH 1.0.

**Figure 2:**
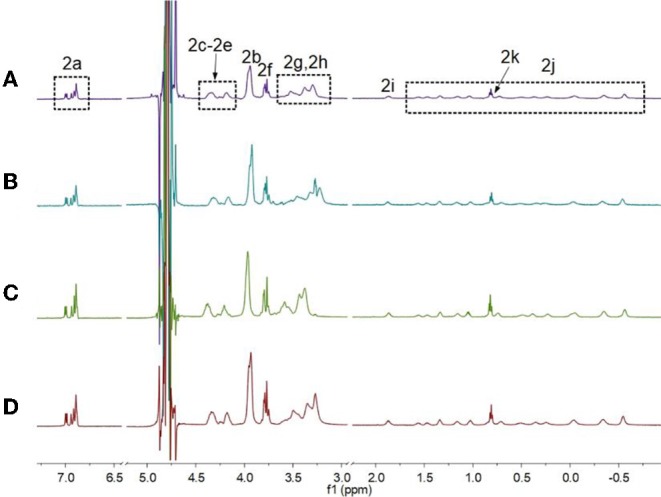
Partial ^1^H NMR spectra of **H2** (600 MHz, D_2_O, room temperature) at different concentrations when the pH-value of the solutions was 3.0: **(A)** 0.500 mM, **(B)** 1.00 mM, **(C)** 2.50 mM, and **(D)** 5.00 mM.

First, ^1^H NMR spectroscopy experiments were performed to study the pH-induced transition between **H1** and **H2**. Owing to the poor solubility of **H1** in water, the ^1^H NMR experiments of **H1** was done in the mixture of D_2_O and CD_3_CN (Figures 1A–C). With the decrease in the pH-value of the aqueous solution of **H1**, the morpholine groups on **H1** were protonated, and **H1** changed into **H2**. Therefore, the peaks corresponding to the protons on **H2** were quite different from that of **H1**. As shown in Figures 1D–G, the signals for protons H_2g_ and H_2h_ appeared in upfield comparing to the protons H_1g_ and H_1h_ on **H1**. In addition, the signals for the protons H_2i_-H_2k_ on the alkyl chain of **H2** appeared in upfield and splitted compared to the related protons on **H1**. This phenomenon was because the alkyl chain threaded into the cavity of **H2**, forming cyclic oligomers. To investigate whether the specific structures of **H2** occur only at low concentrations, concentration-dependent ^1^H NMR experiments were carried out. As shown in Figure 2, with the increase in the concentration from 0.500 to 5.00 mM, the peaks related to **H2** did not show changes. In addition, at first, we assumed that **H2** can also act as monomers for supramolecular polymers like other systems (Shi et al., 2014). However, **H2** showed poorer solubility in water than other monomers. That is why the concentration-dependent ^1^H NMR experiments were only done in the range of 0.500–5.00 mM. Therefore, the conclusion can be drawn that **H2** formed [2]daisy chains in water, which did not changed with its concentration (Zhang et al., 2011).

### 2D Nuclear Overhauser Effect Spectroscopy Study

**Figure 3:**
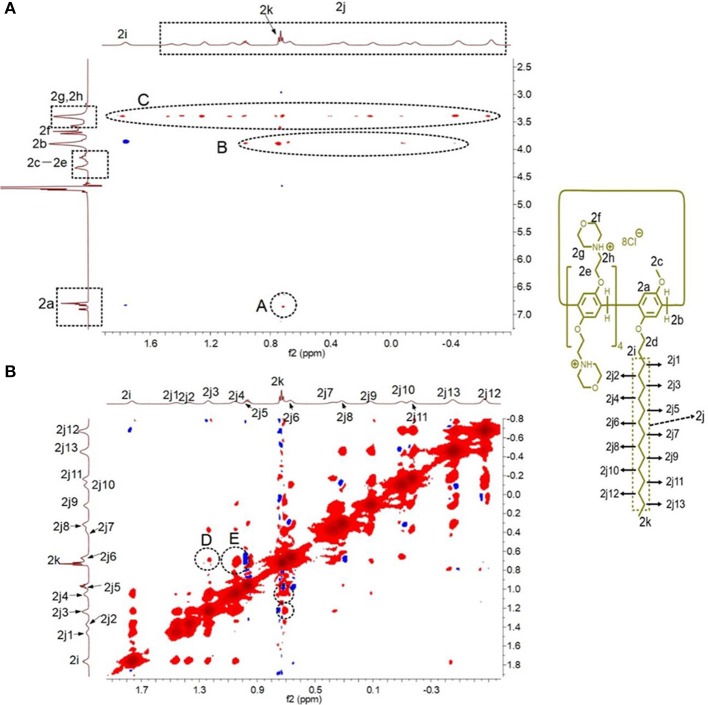
Partial 2D NOESY spectra (600 MHz, D_2_O, room temperature) of **H2** (5.00 mM): **(A)** the NOE correlation signals between the pillar[5]arene ring and the alkyl chain were marked; **(B)** the NOE correlation signals were also observed between alkyl chains were marked.

2D nuclear overhauser effect spectroscopy (NOESY) was performed to monitor the formation of the [*c*2]daisy chain based on **H2**. As shown in Figure 3, NOE correlation signals were observed between the protons H_2a_ on the phenyl rings and H_2k_ on the alkyl chain (A), between H_2b_ on the methylene bridge and H_2k_-H_2j_ on the alkyl chain (B), and between protons H_2g_, H_2h_ on the morpholine groups and H_2i_-H_2k_ on the alkyl chain (C), suggesting that the alkyl chain thread into the cavity of **H2**. In addition, NOE correlation signals were also observed between alkyl chains, including the signals between protons H_2j3_ and H_2k_ (D) and between protons H_2j4_ and H_2k_ (E), confirming the formation of the [*c*2]daisy chain based on **H2**.

### Critical Aggregation Concentration Determinations

**Figure 4:**
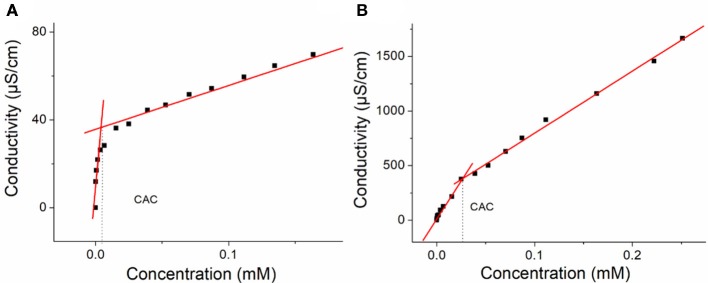
**(A)** The concentration-dependent conductivity of **H1**. The critical aggregation concentration (CAC) was determined to be 3.69 × 10^−6^ M and **(B)** the concentration-dependent conductivity of the **H2**. The CAC was determined to be 2.67 × 10^−5^ M.

The CACs of **H1** and **H2** were measured. As shown in Figure 4, the CACs of **H1** and **H2** were measured to be 3.69 × 10^−6^ M and 2.67 × 10^−5^ M, respectively, using the concentration-dependent conductivity measurements.

### Transmission Electron Microscopy and Atomic Force Microscopy Investigations

**Figure 5:**
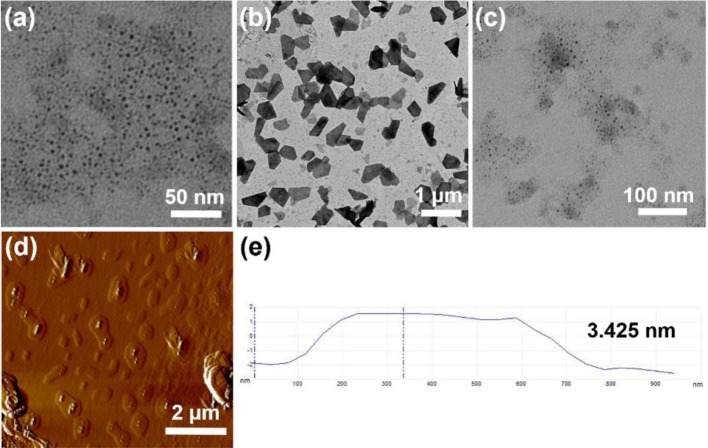
**(a)** TEM image of **H1** (1.00 × 10^−4^ M) aggregates in water; **(b)** TEM image of a after addition of hydrochloric acid; **(c)** TEM image of **(b)** after addition of sodium hydroxide; **(d)** AFM image of **(b)**; **(e)** measured thickness of **(d)**.

The self-assembly morphologies in water were investigated by TEM and AFM. As shown in Figure 5a, **H1** formed micelles with an average diameter of ~6 nm, which was near to the length of two **H1** molecules. After adding hydrochloride acid to the solution of **H1** to adjust the pH-value to 4.0, **H1** turned into **H2**, the micelles changed into nanosheets (Figure 5b). After further addition of sodium hydroxide to the solution of **H2**, the nanosheets turned back to micelles (Figure 5c). AFM experiments were also carried out to investigate the self-assembled morphology by **H2**. As shown in Figures 5d,e, the nanosheet morphology was confirmed and the wall thickness was ~3.425 nm from AFM results, which was about the extended length of the [*c*2]daisy chain, suggesting that the nanosheets had a bilayer wall.

## Conclusion

In summary, a pillararene-based macrocyclic amphiphile **H1** was prepared. **H1** can act as a single-chain amphiphile and self-assembled into micelles in water. After changing the pH of the solution of **H1** to below 5.0, the single-chain amphiphiles turned into [*c*2]daisy chain-based bola-type supramolecular amphiphiles. As a result, the micelles turned into nanosheets when self-assembling in water. This pH-induced transition between macrocyclic single-chain amphiphile and [*c*2]daisy chain-based bola-type supramolecular amphiphiles based on pillararenes was first reported, providing a new strategy to tailor the structure and self-assembly property of macrocyclic amphiphiles. The corresponding pH-responsive self-assembly system provides a promising candidate for advanced material such as controlled release, drug delivery systems, and surface modification.

## Data Availability Statement

Compound characterization, full synthetic details for this study are included in the article/Supplementary Material.

## Author Contributions

PW, RW, and DX made contributions to the experiments. All authors extensively discussed the results. The paper was written by PW. All authors reviewed the manuscript. All authors extensively discussed the results, reviewed the manuscript, and approved the final version of the manuscript to be submitted.

### Conflict of Interest

The authors declare that the research was conducted in the absence of any commercial or financial relationships that could be construed as a potential conflict of interest.
