# Supplementary material for: pH-Induced Transition Between Single-Chain Macrocyclic Amphiphile and [c2]Daisy Chain-Based Bola-Type Amphiphile and the Related Self-Assembly Behavior in Water
Source: Front Chem. 2020 Jan 24;7:894. doi: 10.3389/fchem.2019.00894 (PMC6992661; doi:10.3389/fchem.2019.00894)
Supplement: Supplementary file 1 [file Data_Sheet_1.PDF]

pH-Induced Transition between Single-Chain Macrocyclic Amphiphile and [*c*2]Daisy Chain-Based Bola-Type Amphiphile and the Related Self-Assembly Behavior in Water

Pi Wang[1*], Ruihuan Wang[1], Danyu Xia[2*]

[1]Ministry of Education Key Laboratory of Interface Science and Engineering in Advanced Materials, Taiyuan University of Technology, Taiyuan 030024, China.

[2]Scientific Instrument Center, Shanxi University, Taiyuan 030006, P. R. China.

Supplementary Material

1. *Materials and Methods* *S2*
2. *Syntheses of* ***H1*** *S3*

References *S6*

1. Materials and methods

All reagents were commercially available and used as supplied without further purification. Compounds **a**[S1] were prepared according to published procedures. NMR spectra were recorded with a Bruker Avance DMX 600 spectrophotometer or a Bruker Avance DMX 400 spectrophotometer. Low-resolution electrospray ionization mass spectra were recorded with a Bruker Esquire 3000 Plus spectrometer. High-resolution mass spectrometry experiments were performed with a Waters UPLC H-Class QDA instrument. The melting points were collected on a SGW X-4 automatic melting point apparatus. The determination of the critical aggregation concentration (CAC) values was carried out on a FE38 instrument. Transmission electron microscopy investigations were carried out on a JEM-1200EX instrument. Atomic force microscopy experiments were performed by a Bruker Multi-Mode 8.0 instrument.

2. *Syntheses of **H1***

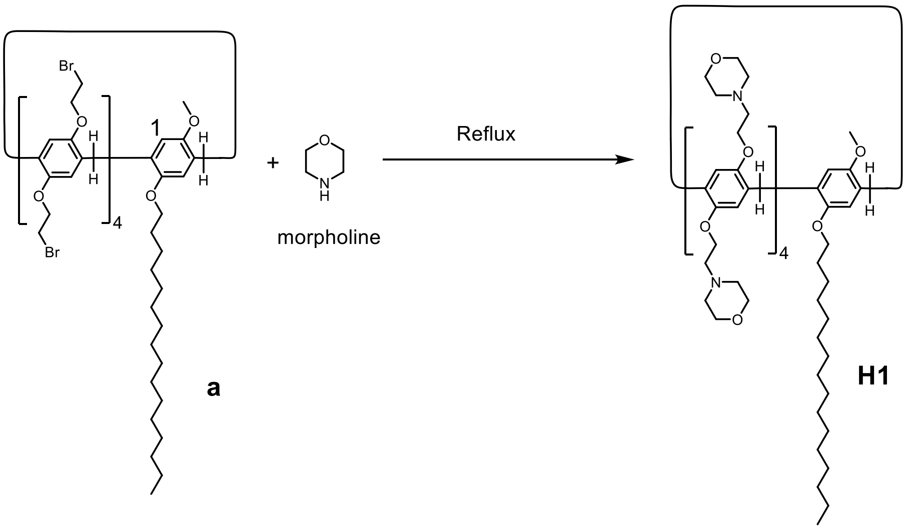

Scheme S1 Synthetic route to **H1**

Compound **a** (1.08 g, 0.622 mmol) and morpholine (0.566 g, 6.50 mmol) were added to acetonitrile (10.0 mL). The solution was refluxed overnight. Then the crude product was purified by a silica gel column using dichloromethane as eluent. (0.421 g, 38 %). Mp: 75.0–77.0 ^{o}C. The ^{1}H NMR spectrum of **H1** is shown in Fig. S1. ^{1}H NMR (400 MHz, $CDCl_3$, 298 K) δ (ppm): 6.85 (s, 10H), 4.14–4.09 (m, 8H), 3.98–3.92 (m, 9H), 3.75–3.73 (m, 47H), 2.88–2.75 (m, 16H), 2.62–2.61 (m, 32H). 1.95-1.86 (m, 2H), 1.84 – 1.72 (m, 2H), 1.56 – 1.47 (m, 2H), 1.41 – 1.32 (m, 2H), 1.16 – 1.07 (m, 20H), 0.85 (t, J = 8.0 Hz, 3H). The ^{13}C NMR spectrum of **1** is shown in Fig. S2. ^{13}C NMR (100 MHz, $CDCl_3$, 298 K) δ (ppm): 149.47, 148.84, 148.77, 127.82, 127.65, 127.50, 127.45, 127.06, 114.32, 114.18, 112.90, 67.69, 65.99, 65.99, 65.81, 57.23, 54.93, 53.27, 52.34, 30.91, 28.78, 28.72, 28.64, 28.59, 28.44, 28.39, 28.35, 28.28, 25.13, 21.68, 13.13. HRESIMS is shown in Fig. S3: *m*/*z* calcd for $[M + 2H + e]^{+}$ $C_{102}H_{158}N_8O_{18}$, 1783.16941, found 1783.16784, error −0.9 ppm; *m*/*z* calcd for $[M + 3H + e]^{2+}$ $C_{102}H_{159}N_8O_{18}$, 892.08862, found 892.08527, error −3.8 ppm.

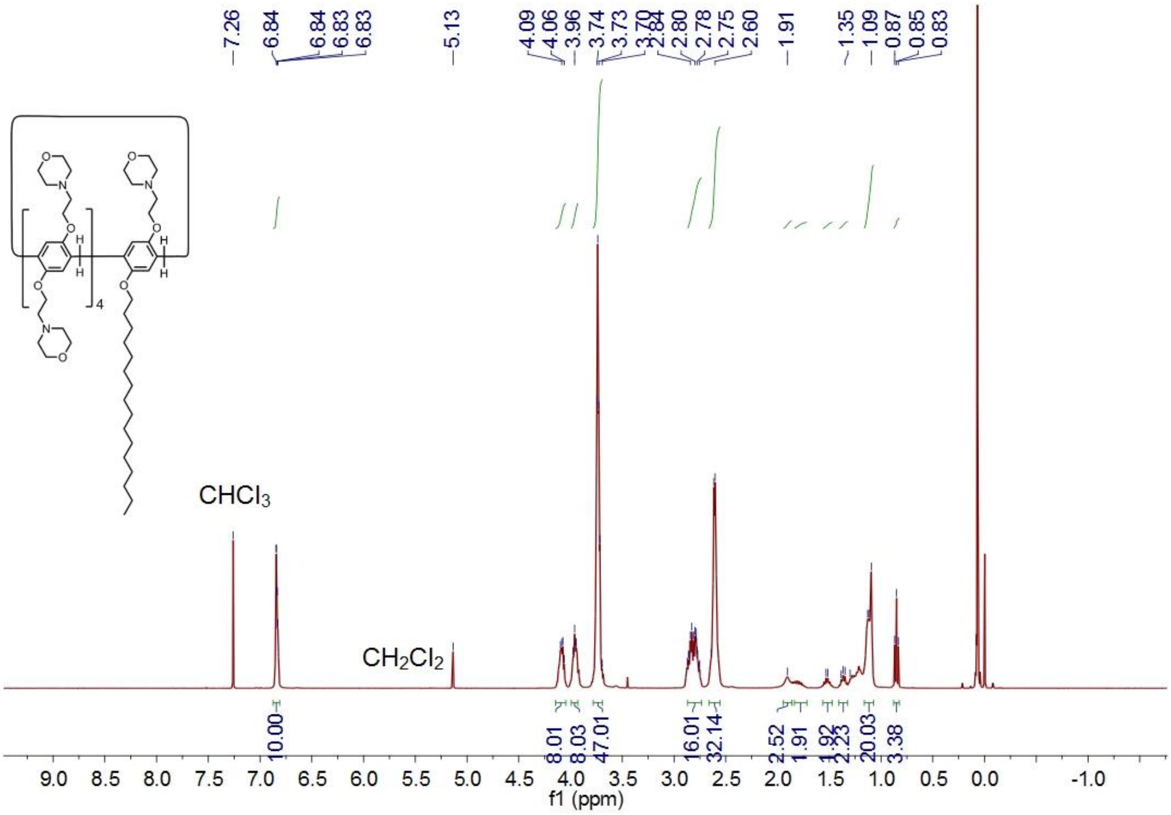

Figure S1. ^{1}H NMR spectrum (400 MHz, $CDCl_3$, room temperature) of **H1**.

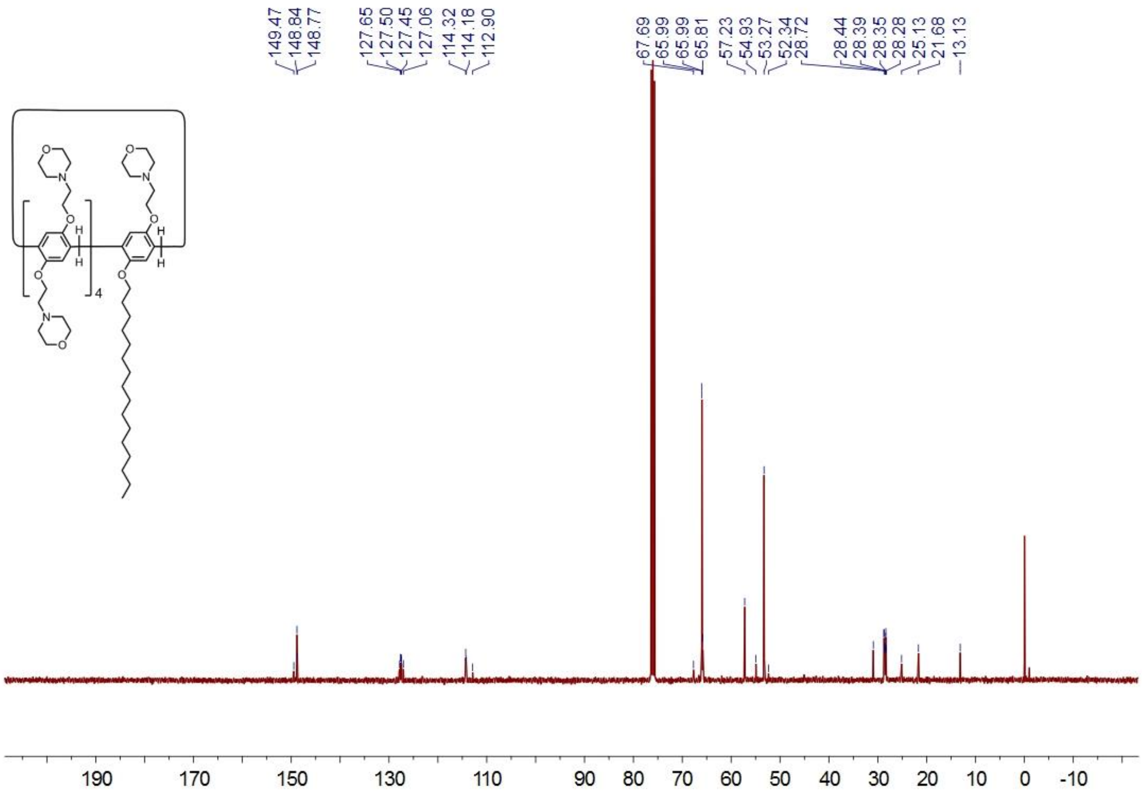

Figure S2. ^{13}C NMR spectrum (100 MHz, $CDCl_3$, room temperature) of **H1**.

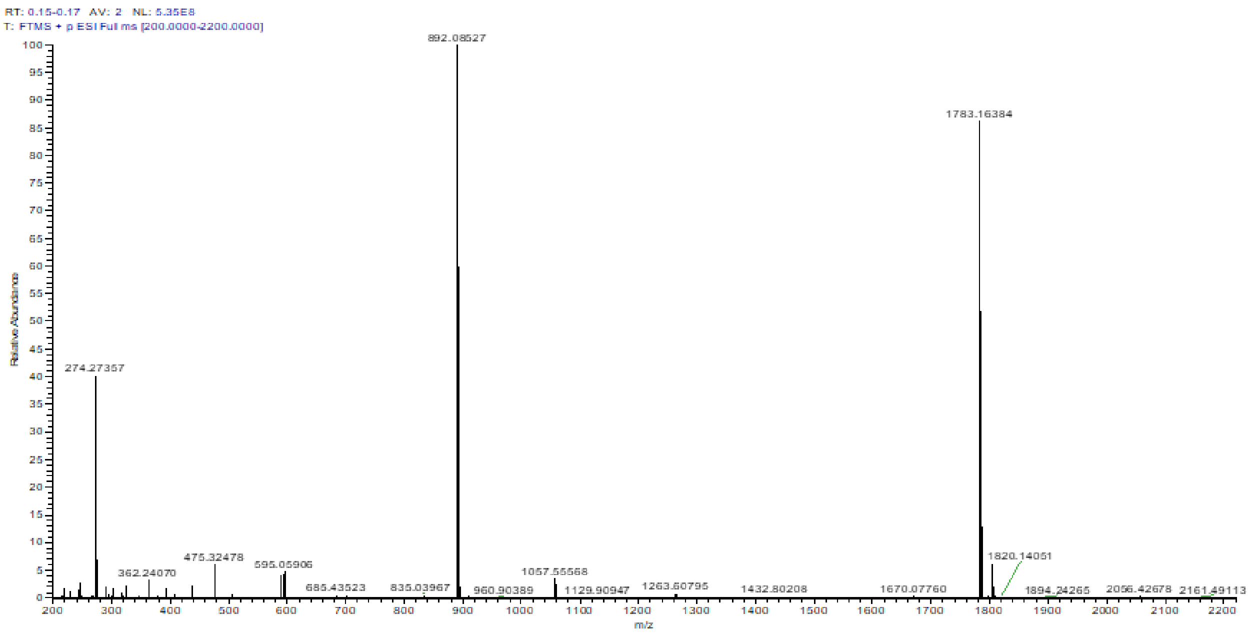

Figure S3. High-resolution mass electrospray ionization mass spectrum of **H1**. Main peak: *m/z* 892.08527 [M + 3H + e]$^{2+}$ (100 %); *m/z* 1783.16384 [M + 2H + e]$^{+}$ (87 %)

References:

S1. Shi, B.; Xia, D.; Yao, Y., *Chem. Commun.*, 2014, **50**, 13932–13935.
